# Platelets, Biomarkers of Coagulation and Fibrinolysis, and Early Coronary Stent Thrombosis

**DOI:** 10.3390/jcm14010056

**Published:** 2024-12-26

**Authors:** Lukas Galli, Alexander Sator, Stephanie Schauer, Konstantin Bräu, Johannes Bernhard, Christian Hengstenberg, Clemens Gangl, Rayyan Hemetsberger, Christian Roth, Rudolf Berger, Konstantin A. Krychtiuk, Walter S. Speidl

**Affiliations:** 1 Division of Cardiology, Department of Internal Medicine II, Medical University of Vienna, 1090 Vienna, Austria; lukas.galli@meduniwien.ac.at (L.G.); n11715688@students.meduniwien.ac.at (A.S.); stephanieschauer@icloud.com (S.S.); braeu.konstantin@icloud.com (K.B.); johannes.bernhard@meduniwien.ac.at (J.B.); christian.hengstenberg@meduniwien.ac.at (C.H.); clemens.gangl@meduniwien.ac.at (C.G.); rayyan.hemetsberger@meduniwien.ac.at (R.H.); walter.speidl@meduniwien.ac.at (W.S.S.); 2 Ludwig Boltzmann Institute for Cardiovascular Research, 1090 Vienna, Austria; 3 Department of Internal Medicine I, Cardiology and Nephrology, Hospital of St. John of God, 7000 Eisenstadt, Austria; rudolf.berger@bbeisen.at

**Keywords:** coronary stent thrombosis, D-Dimer, platelet count, fibrinogen, coagulation, inflammation

## Abstract

**Background/Objectives**: Acute stent thrombosis (ST) is a rare yet severe complication following percutaneous coronary intervention (PCI). Herein, we investigated the possible association between routinely available coagulation and fibrinolysis markers with early ST. **Methods**: Within a single-center registry, we investigated the association between the preprocedural platelet count, plasma levels of fibrinogen and D-Dimer, and the incidence of early ST in the first 30 days after PCI. **Results**: Out of 10,714 consecutive patients who underwent PCI using drug-eluting stents (DESs), the preprocedural platelet count, fibrinogen, and D-Dimer measurements were available in 6337, 6155, and 956 patients, respectively. Fifty-eight patients (0.92%) experienced an early ST within 30 days after PCI. Compared with those without ST, patients with early ST showed significantly elevated preprocedural platelet counts (*p* < 0.05) and fibrinogen levels (*p* < 0.05). D-Dimer levels were not associated with early ST. Patients in the fifth quintile of platelet count had a significantly increased risk for early ST (HR 2.43; 95% CI 1.43–4.14; *p* = 0.001) compared with patients in the lower four quintiles. In addition, patients in the fifth quintile of fibrinogen also had a significantly increased risk for early ST (HR 1.86; 95% CI 1.07–3.26; *p* < 0.05) compared with patients in the lower four quintiles. These associations were independent of clinical risk factors, the number of stents, the presence of acute coronary syndromes, and white blood cell count. **Conclusions**: Preprocedural platelet counts and fibrinogen plasma levels can identify patients at elevated risk of early ST after implantation of DESs in addition to procedure-level and device-related risk factors.

## 1. Introduction

Annually, over 1 million percutaneous coronary interventions (PCIs) are carried out in the United States and Europe combined, both for the treatment of flow-limiting lesions in stable ischemic heart disease (SIHD) and, most importantly, as a life-saving treatment in a patient with acute thrombotic occlusion of an epicardial coronary artery. Stent thrombosis (ST) is a rare but severe and dangerous complication of PCI and is associated with a high rate of mortality and morbidity. ST can occur early (<30 days), late (30 days up to 1 year), and very late (>1 year) after PCI intervention, while early ST seems to have the highest in-hospital mortality [1,2]. Based on the Academic Research Consortium-2 (ARC-2), ST is identified as silent ST (with the absence of clinical signs and symptoms), as probable ST (with MI and acute ischemia in the territory of the previously implanted stent without angiographic confirmation), and definite ST (with angiographic or pathological confirmation).

Several predictors and risk factors for ST have been identified based on patient-, lesion-, device-, and procedure-related characteristics [3,4,5]. While improvements in stent technology, PCI techniques, and drug therapy have reduced ST risk [6], residual risk remains. Reliable preprocedural and periprocedural (bio-)markers of elevated risk of ST, which might trigger more aggressive medical treatment, are scarce and of great clinical need [7]. Biomarkers reflecting activation of the coagulation system are biologically plausible candidates. In a contemporary registry-based analysis, patients treated for acute myocardial infarction (AMI), as compared with patients undergoing PCI for SIHD, were at higher risk of ST, and most ST occurred in patients with high platelet reactivity [8]. In the immediate and long-term aftermath of an AMI, many patients experience an ongoing activation of the coagulation system and thrombin-mediated platelet activation, all mechanisms implicated in elevated risk of thrombotic complications and attenuated with additional anticoagulation treatment [9,10,11]. Significantly fewer data are available for patients undergoing PCI for SIHD.

The aim of this study was therefore to investigate whether routinely available laboratory parameters reflecting ongoing coagulation and/or fibrinolysis such as platelet count, fibrinogen, and D-Dimer may identify patients at high risk of early ST after PCI.

## 2. Materials and Methods

The coronary catheter laboratory registry of the Medical University of Vienna (CCLD-MUW) is a registry that includes all consecutive patients > 18 years undergoing coronary angiography and PCI at the General Hospital of Vienna/Medical University of Vienna, Austria. Out of the total cohort of 11,337 patients, 10,624 (93.7%) underwent PCI with DES for both AMI and SIHD [12]. The study was in line with the Declaration of Helsinki and was approved by the ethics committee of the Medical University of Vienna. As this was a retrospective study, informed consent is not required according to local ethics committee.

Data and endpoint collection are described elsewhere [13]. The primary endpoint was specified as confirmed early stent thrombosis occurring within 30 days post-PCI, determined according to the 2018 Academic Research Consortium-2 (ARC-2) consensus, which provides standardized criteria for defining endpoints in coronary intervention studies [2]. ARC-2 defines definite early stent thrombosis as stent thrombosis confirmed by coronary angiography or autopsy within the first 30 days after the initial PCI (acute, 0–24 h; subacute, >24 h to 30 days) [2].

Preprocedural (within 24 h prior to angiography) measurements of platelet count, fibrinogen, and D-Dimer were performed by the central laboratory of the General Hospital of Vienna, according to the team’s discretion.

### Statistical Analysis

Categorical variables were summarized as counts or percentages and compared by the χ^2^ or Fisher’s exact test as appropriate. Continuous variables were expressed as the median (interquartile range). Unpaired variables were compared using the Mann–Whitney test for two samples and the Kruskal–Wallis test for multiple samples. Paired variables were compared using the Wilcoxon rank-sum test. Patients were divided into quintiles according to preprocedural platelet count, fibrinogen, and D-Dimer measurement. Kaplan–Meier analysis with the log-rank test was used to assess the impact of hemostatic markers on stent thrombosis. Cox proportional hazard regression analysis was conducted to evaluate the risk of stent thrombosis in patients in the fifth quintile compared with those in the lower four quintiles of the respective markers. Variables that were clinically relevant or showed an imbalance between patients with and without stent thrombosis, as indicated by a *p*-value < 0.1 in univariate analysis, were included in the study. Precision–recall curves were utilized to evaluate the model performance due to the imbalanced population. Optimal cut-off values were determined using the Youden index, and c-statistics were calculated to assess the discriminative ability of these cut-off values. Two-sided *p*-values of <0.05 indicated statistical significance. The statistical analyses were conducted using SPSS 29.0 (IBM Corporation, Armonk, NY, USA).

## 3. Results

### 3.1. Baseline Characteristics

Out of 10,624 patients who underwent PCI with DES implantation, preprocedural data were available for platelet count in 6337 cases, fibrinogen in 6155, and D-Dimer in 956 (see supplemental Table S1 for baseline characteristics of the whole study cohort as compared with patients with available biomarkers). The median age of patients was 64 (IQR 54–72) years, and 1770 (27.9%) were female (Table 1). A total of 3167 (50%) of the patients had acute coronary syndrome (ACS), and 3170 (50%) were treated for SIHD. Of the 6337 patients with at least one marker available, 58 (0.92%) had a definite early ST within 30 days. The clinical risk factors of smoking (3026, 48.2% vs. 27, 46.6%; *p* = 0.735), diabetes (1570, 25% vs. 13, 22.4%; *p* = 0.897), and arterial hypertension (4490, 71.5% vs. 39, 67,2%; *p* = 0.474) were not associated with early ST. In contrast, the presence of ACS (*p* < 0.001), as well as multivessel disease (*p* = 0.031) and the number of stents (*p* = 0.001), was associated with increased risk for early ST (Table 1).

### 3.2. Preprocedural Platelet Counts, Biomarkers of Coagulation, Fibrinolysis, and Definite Early Stent Thrombosis

**Figure 1:**
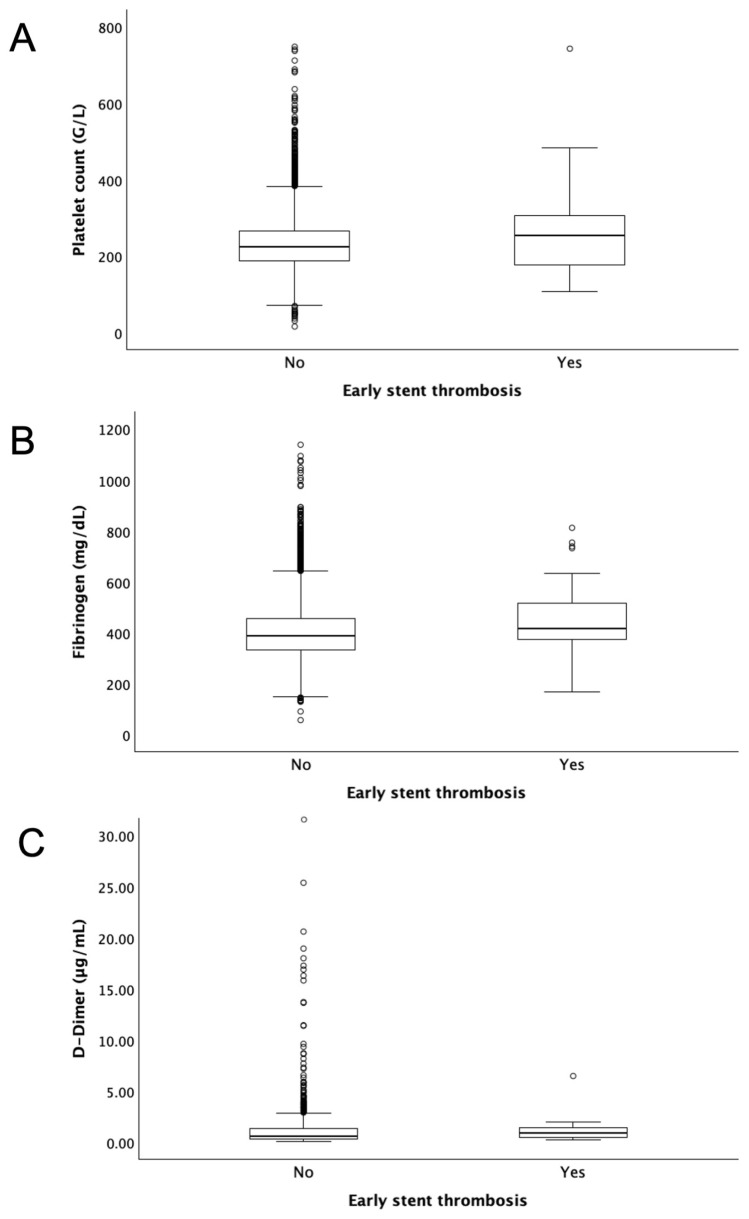
Preprocedural coagulation parameters and early stent thrombosis. Preprocedural platelet count (**A**), plasma levels of fibrinogen (**B**), and plasma levels of D-Dimer (**C**) in patients with and without definite early (<30 days) stent thrombosis.

The preprocedural platelet count was significantly higher in patients with early ST (255 IQR 178–307 G/L; *n* = 58) as compared with patients without early ST (224 IQR 188–266 G/L; *p* < 0.05; *n* = 6279; Figure 1A). In addition, patients with early ST showed higher preprocedural plasma levels of fibrinogen (416 IQR 374–515 mg/dL; *n* = 57) as compared with patients without ST (387 IQR 333–457 mg/dL; *p* < 0.05; *n* = 6098; Figure 1B). In contrast, D-Dimer levels were not different in patients with (*n* = 17) and without (*n* = 939) early ST (0.88 IQR 0.44–1.43 µg/mL vs. 0.60 IQR 0.33–1.36 µg/mL; *p* = 0.43; Figure 1C). Patients with platelet counts in the fifth quintile (>278 G/L) demonstrated a significantly higher risk of early ST (HR 2.43; 95% CI 1.43–4.14; *p* = 0.001) compared with those in the lower four quintiles. Likewise, patients in the fifth quintile of fibrinogen (>482 mg/dL) experienced a significantly increased risk for early ST (HR 1.86; 95% CI 1.07–3.26; *p* < 0.05) as compared with patients in the lower four quintiles. These associations were independent of age, sex, acute coronary syndromes, the number of stents, and leukocyte count (Table 2). In contrast, the highest quintile of D-Dimer plasma levels (>1.65 µg/mL) was not associated with early ST (HR 1.17; 95% CI 0.34–4.08; *p* = 0.80).

### 3.3. Preprocedural Platelet Count in Combination with Fibrinogen and Definite Early Stent Thrombosis

**Figure 2:**
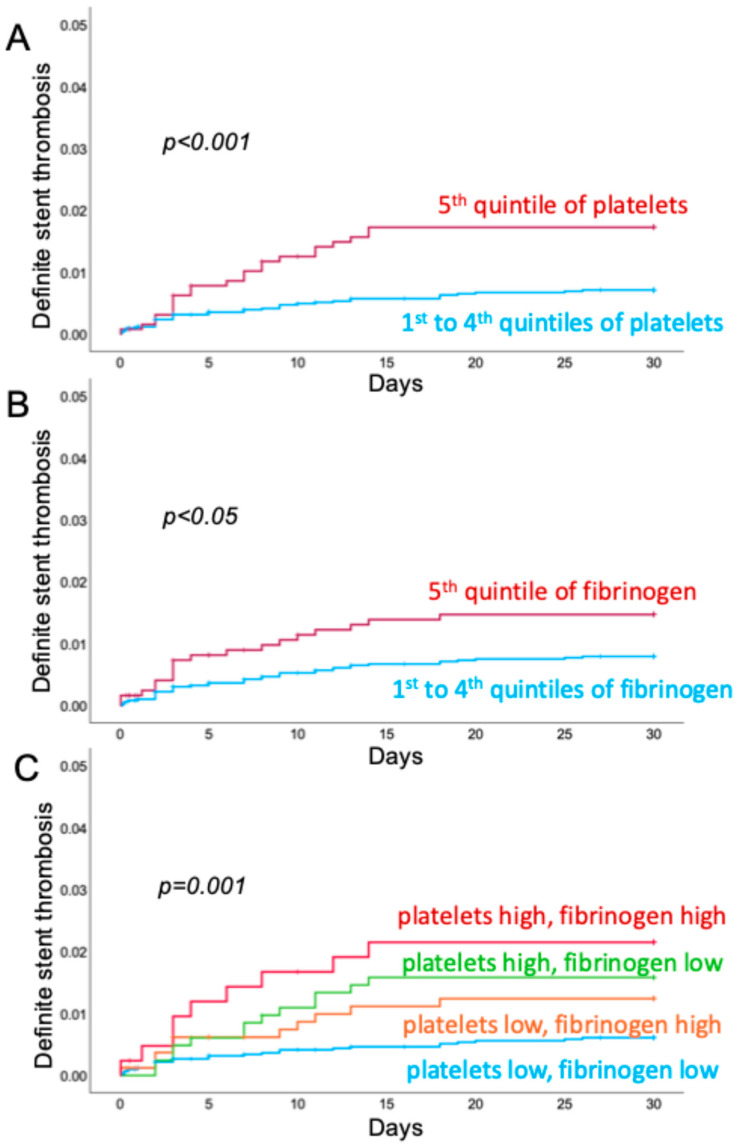
Kaplan–Meier curves for early stent thrombosis according to quintiles of coagulation parameters. Patients were stratified according to preprocedural platelet count (**A**) and plasma levels of fibrinogen (**B**) above and below the fifth quintile. Patients with platelet count and fibrinogen in the fifth quintile (platelets high and fibrinogen high) showed a markedly increased rate of definite, early (<30 days) ST. Patients with platelet count and fibrinogen in the four lower quintiles (platelet low, fibrinogen low) showed the lowest rate of ST (**C**). The *p*-value was calculated by log-rank test.

Figure 2A,B show Kaplan–Meier curves for the fifth versus the lower quintiles of the preprocedural platelet count and plasma levels of fibrinogen, respectively. Figure 2C shows Kaplan–Meier curves for the combination of preprocedural platelet count and fibrinogen levels. Interestingly, in patients with both platelet count and fibrinogen in the fifth quintile, the rate of definite early stent thrombosis was severely elevated at 2.1% when compared with patients with preprocedural platelet count and fibrinogen levels below the fifth quintile (0.6%; log-rank *p* < 0.001). Kaplan–Meier curves for patients stratified according to SIHD or the presence of ACS are presented in the Supplementary Materials (Supplemental Figures S1 and S2).

### 3.4. Cut-Off Values and C-Statistics for Platelet Count and Fibrinogen to Predict Definite Early Stent Thrombosis

Using the Youden index to determine optimal cut-off values, a preprocedural platelet count of 285 G/L was identified (c-statistic = 0.60; *p* = 0.014), along with a fibrinogen level of 376 mg/dL (c-statistic = 0.59; *p* = 0.010) suggesting moderate discriminative ability for predicting the occurrence of early ST. Kaplan–Meier curves for patients above and below the determined cut-off values for platelet count and fibrinogen are presented as Supplemental Figure S3, and precision-recall curves for platelet count and fibrinogen are presented as Supplemental Figure S4.

### 3.5. Early Stent Thrombosis, Preprocedural Markers of Coagulation, and Mortality

Definite early ST was present in 58 of 6.337 patients (0.92%) within 30 days. The 30-day all-cause mortality was 22.4% in patients who experienced an ST compared with 3.4% in those without ST (*p* < 0.00001). Out of the preprocedural markers of coagulation, the fifth quintile of preprocedural plasma levels of fibrinogen (log-rank *p* = 0.002) and D-Dimer (log-rank *p* = 0.032) were associated with increased thirty-day mortality. In contrast, preprocedural platelet count did not predict survival (log-rank *p* = 0.52; Supplemental Figure S5).

## 4. Discussion

In this observational analysis, which included 6337 patients who underwent PCI for ACS or SIHD, 58 (0.92%) patients experienced a definite early ST within 30 days. The occurrence of ST was associated with a drastic, nearly six-fold elevated mortality risk as compared with patients without ST. We found that preprocedural platelet counts and fibrinogen levels were significantly higher in patients experiencing an early ST. Patients with elevated platelet counts or fibrinogen in the fifth quintile had HRs of 2.43 and 1.86 for early ST, respectively, compared with patients in the lower quintiles. Furthermore, these associations were independent of other risk factors like sex, hypertension, the presence of ACS, and the number of stents. Patients with elevated platelet count and fibrinogen levels in the fifth quintiles showed a more than three-fold elevated rate of early ST of 2.1%. In contrast, D-Dimer levels were not associated with early ST in our cohort.

Stent thrombosis is a rare yet serious complication of PCI, associated with a high mortality rate ranging from 10% to 33% [3,6,14]. In our cohort, stent thrombosis was linked to a significantly higher 30-day mortality rate of 22.4%, compared with just 3.4% in patients without stent thrombosis. Thus, preventing stent thrombosis should be a primary focus in both clinical practice and research.

Ample evidence exists regarding on-treatment platelet reactivity and risk of coronary stent thrombosis after PCI [15,16,17]. However, establishing (preprocedural) risk factors and having readily available (bio-)markers reflecting ST risk before PCI and initiating antithrombotic therapy would be of utmost clinical interest. This could help in decision-making when choosing the revascularization modality and strategy; e.g., a very high risk for ST could favor coronary artery bypass grafting (CABG) over PCI. In addition, an increased risk for ST could influence the PCI technique, e.g., reducing the number and length of stents, using intravascular imaging with either intravascular ultrasound or optical coherence tomography (OCT) to guide implantation of coronary stents and performing extensive post-dilation techniques to optimize stent deployment, which all have been shown to reduce the risk of ST [18,19,20]. In particular, in STEMI patients at high risk for ST, OCT could enable precise lesion assessment, optimal stent sizing, and verification of stent deployment efficacy thereby reducing the risk of stent malapposition leading to ST. Recent meta-analyses demonstrated that OCT-guided PCI significantly enhances the safety and effectiveness of revascularization, leading to reduced risks of ST [21].

Several scores have been developed to predict ST after PCI [22,23,24]. All of these scores have in common that clinical factors like the presence of acute coronary syndromes, prior revascularization, diabetes mellitus, and smoking were associated with increased risk of ST. The score developed by Dangas et al. also included the preprocedural platelet count [23], which aligns well with our study observation. The ADAPT-DES study investigated whether the platelet count was associated with on-treatment platelet reactivity. Interestingly, lower levels of platelet count were strongly associated with higher residual platelet activity. However, higher platelet counts significantly predicted thrombotic events after PCI despite lower residual platelet activity [25]. In line with the previous literature, patients with STEMI were at highest risk for the development of early ST. Stent thrombosis often results from incomplete endothelialization, malapposition, delayed arterial healing, or hypersensitivity reactions to stent components. These factors, compounded by the hypercoagulable state often seen in STEMI patients, exacerbate the risk of thrombotic events. STEMI itself triggers systemic inflammation, platelet activation, and coagulation cascade activation, further increasing the propensity for stent thrombosis [3,5,26].

In contrast to the role of platelets in the development of ST, data regarding the role of the coagulation system are sparse [11,27]. A case-control study showed that ST patients showed a hypercoagulable state with increased thrombin generation by the contact activation pathway [28]. In addition, patients who developed ST demonstrated abnormal plasma clot characteristics (e.g., permeability, turbidity, and lysis time) compared with patients who did not [29]. Although fibrinogen has been linked to outcomes in patients with SIHD [30], it has not yet been shown to predict ST. In our study, patients with elevated fibrinogen were at significantly increased risk for early ST. A beneficial effect of anticoagulation therapy, in addition to antiplatelet therapy, in patients after acute MI has been observed [9,10,11]. Thus, an overactive coagulation system might contribute to the risk for ST.

We have recently shown that periprocedural inflammatory activation is associated with an increased risk for early ST [13]. Interestingly, fibrinogen is an acute-phase protein, and plasma fibrinogen levels are increased during acute inflammation [31]. In addition, platelet count might be increased during inflammatory conditions [32]. Therefore, we adjusted for the inflammatory parameter leukocyte count, which did not significantly attenuate the association between platelet count or fibrinogen with ST. Several other factors have been shown to increase the risk of ST. One of these is stress hyperglycemia, which has been shown to increase the risk of ST in patients with STEMI [33], and the stress hyperglycemia ratio (SHR) was associated with worse short- and long-term outcomes in patients with acute MI [34]. However, we were not able to include the SHR in our multivariate analysis, as glucose values were not available retrospectively.

Early ST was associated with a 6.5-fold increased risk of 30-day mortality. In addition, elevated plasma levels of fibrinogen and D-Dimer were associated with decreased survival. Interestingly, while the platelet count was a strong predictor of early ST, it did not predict outcomes in this study. This could be explained by the fact that the platelet count, while essential for thrombus formation, may not fully capture the broader systemic processes influencing survival, such as the extent of ischemic injury, inflammatory burden, or the interplay of other hemostatic factors. These findings suggest that the prognostic impact of platelet count might be overshadowed by more dynamic markers like fibrinogen and D-dimer, which reflect ongoing thrombotic and inflammatory processes more directly associated with mortality risk [11].

In recent years, individualization of the intensity and duration of antiplatelet therapy after coronary stenting has gained attention. The selection of an optimal dual antiplatelet therapy (DAPT) regimen remains a dynamic challenge, requiring careful consideration of the interplay between antiplatelet agents, patient-specific factors, and procedural char-acteristics. While guidelines offer general frameworks, the evolving trend is toward a more individualized approach. These data highlight the potential role of preprocedural biomarkers, such as platelet count and fibrinogen levels, in tailoring the intensity and duration of antiplatelet therapy. These markers may aid in stratifying patients based on their risk of ST, enabling clinicians to fine-tune therapy to achieve better outcomes while minimizing bleeding risks [35].

### Limitations

Several limitations of this study need to be mentioned. Of the 10,714 patients of this registry who underwent PCI with DES, only 6337, 6155, and 956 patients had platelet count, fibrinogen, and D-Dimer measurements, respectively. Due to the lower number of patients with preprocedural D-Dimer, we cannot rule out type I errors due to the limited power of the analysis. In addition, we must consider the possibility of accidentally selecting high-risk patients based on the availability of preprocedural laboratory values. In addition, we observed a higher percentage of patients with ACS in the early ST cohort, known for having a higher risk for early ST than SIHD patients [8]. However, while this could be a confounding factor, our findings could provide insight into the mechanisms involved in the increased ST risk in patients with ACS. Patients with elevated biomarkers of coagulation were consistently shown to be at increased risk for major adverse cardiac events after the acute ACS phase [11]. Another limitation is that blood sampling was conducted within a 24 h window prior to PCI. This timing introduces potential variability, particularly in patients with ACS, as levels of fibrinogen and D-dimer are known to fluctuate significantly during this period. In contrast, such variability is less pronounced in patients with SIHD, where these biomarkers tend to remain more stable. This should be integrated into the design and planning of future prospective studies. In addition, we had no functional data of the coagulatory system or platelets available. A future prospective study should include platelet functional activity, as well as viscoelastic testing like thromboelastography (TEG) or rotational thromboelastometry (ROTEM) [11]. Another limitation of this study is its reliance on data from a single-center registry, which may limit the generalizability of its findings to broader, more diverse populations. The conclusions drawn from this cohort require external validation through studies conducted in multi-center settings to ensure their applicability across different clinical environments and patient demographics. Additionally, while the use of multivariable modeling aimed to account for potential confounders, the possibility of residual confounding or unmeasured variables influencing the outcomes cannot be entirely excluded. Factors such as variations in operator technique, institutional protocols, or patient characteristics not captured in the registry may still introduce bias, underscoring the need for cautious interpretation of the results.

## 5. Conclusions

In this registry-based, retrospective analysis, elevated preprocedural fibrinogen levels and platelet counts were associated with early ST. Specifically, patients with fibrinogen levels and platelet counts in the fifth quintile experienced an approximately two-fold increase in risk for ST. Beyond established device-related factors like stent type or size and procedural risks such as peri-interventional medication and complications during stent placement, platelet count and fibrinogen levels may represent additional patient-specific risk factors. These could inform the design of future personalized strategies to prevent these uncommon but serious complications.

## Figures and Tables

**Table 1:** Baseline characteristics with at least one biomarker of coagulation or fibrinolysis available.

	No Early ST (*n* = 6279)	Early ST (*n* = 58)	*p*-Value
Age, median (IQR)	63.7 (54.1–72.4)	60.1 (50–72.7)	0.073
Sex			0.518
Male, *n* (%)	4523 (72%)	44 (75.9%)	-
Female, *n* (%)	1756 (28%)	14 (24.1%)	-
BMI, kg/m^2^, median (IQR)	28 (24.7–30.6)	27.7 (24.2–30.5)	0.652
Smoker, *n* (%)	3026 (48.2%)	27 (46.6%)	0.735
Diabetes mellitus, *n* (%)	1570 (25%)	13 (22,4%)	0.897
Hypertension, *n* (%)	4490 (71.5%)	39 (67.2%)	0.474
Atrial fibrillation, *n* (%)	279 (4.4%)	0	
Family history of CAD, *n* (%)	1334 (21,2%)	10 (17.2%)	0.458
Previous myocardial infarction, *n* (%)	1306 (20.8%)	12 (20,7%)	0.984
Previous PCI, *n* (%)	1081 (17.2%)	11 (19%)	0.725
Previous CABG, *n* (%)	496 (7.9%)	2 (3.4%)	0.210
Total cholesterol (mg/dL), median (IQR)	193.0 (161.0–229.0)	185.0 (168.5–216.0)	0.400
HDL (mg/dL), median (IQR)	44.0 (36.0–52.0)	42.0 (32.5–50.0)	0.110
LDL (mg/dL), median (IQR) †	110.0 (84.0–139.0)	91.0 (70.0–120.0)	0.165
Triglycerides (mg/dL), median (IQR)	135.0 (95.0–200.0)	133.5 (89.5–196.75)	0.766
HbA1c (%), median (IQR)	5.9 (5.5–6.4)	5.8 (5.6–6.2)	0.986
Creatinine (mg/dL), median (IQR)	1.02 (0.89–1.2)	1.00 (0.86–1.25)	0.677
Indication for PCI			* < 0.001
SIHD, *n* (%)	3155 (50.2%)	15 (25.9%)	-
ACS, *n* (%)	3124 (49.8%)	43 (74.1%)	-
ACS type (*n* = 3124/*n* = 43)			* < 0.007
STEMI, *n* (%)	1633 (52.3%)	32 (74.4%)	-
NSTEMI, *n* (%)	998 (31.9%)	10 (23.3%)	-
Unstable angina, *n* (%)	493 (15.8%)	1 (2.3%)	-
Number of stents implanted, mean ± SD	1.57 (±1.1)	2.12 (±1.4)	* < 0.001
Stent type			0.430
Early generation DES §, *n* (%)	3455 (55.0%)	35 (60.3%)	-
New generation DES ‡, *n* (%)	2824 (45.0%)	23 (39.7%)	-
No. of coronary vessels diseased			* 0.031
Single vessel disease, *n* (%)	2838 (44.8%)	18 (31.0%)	-
Multivessel disease (>1 vessel), *n* (%)	3436 (54.2%)	40 (69.0%)	-
P2Y12 inhibitor			0.285
No/unknown, *n* (%)	12 (0.2%)	0 (0%)	-
Clopidogrel, *n* (%)	4868 (87.7%)	40 (78.5%)	-
Prasugrel, *n* (%)	496 (9.0%)	9 (17.6%)	-
Ticlopidin, *n* (%)	3 (0.1%)	0 (0%)	-
Ticagrelor, *n* (%)	165 (3.0%)	2 (3.9%)	-

ACS, acute coronary syndrome; CABG, coronary artery bypass graft; BMI, body mass index; SIHD, stable ischemic heart disease; DES, drug-eluting stent; HDL, high-density lipoprotein; LDL, low-density lipoprotein; STEMI, ST-elevation myocardial infarction; IQR, interquartile range; PCI, percutaneous coronary intervention; SD, standard deviation; ST, stent thrombosis. † LDL value calculated with Friedewald formula; * *p* ≤ 0.05 = significant; § early generation DES include sirolimus- and paclitaxel-eluting stents; ‡ new generation DES include everolimus- and zotarolimus-eluting stents.

**Table 2:** Cox regression analyses for preprocedural platelet count, plasma levels of fibrinogen, and early stent thrombosis.

	Hazard Ratio	95% CI	*p*-Value
Platelet count unadjusted			
Quintiles 1–4	1	-	-
Quintile 5	2.43	1.43–4.14	0.001
Platelet count adjusted for age, sex, ACS, and number of stents			
Quintiles 1–4	1	-	-
Quintile 5	2.50	1.45–4.32	<0.001
Platelet count adjusted for age, sex, and leukocyte count			
Quintiles 1–4	1	-	-
Quintile 5	2.20	1.28–3.81	<0.005
Fibrinogen unadjusted			
Quintiles 1–4	1	-	-
Quintile 5	1.86	1.07–3.26	0.029
Fibrinogen adjusted for age, sex, ACS, and number of stents			
Quintiles 1–4	1	-	-
Quintile 5	1.88	1.07–3.31	0.027
Fibrinogen adjusted for age, sex, and leukocyte count			
Quintiles 1–4	1	-	-
Quintile 5	1.93	1.10–3.38	0.022

CI, 95% confidence interval; ACS, acute coronary syndrome.

## Data Availability

The data presented in this study are only available on request from the corresponding author due to ethical reasons.

## Supplementary Materials

The following supporting information can be downloaded at: https://www.mdpi.com/article/10.3390/jcm14010056/s1.
