# Supplementary material for: Platelets, Biomarkers of Coagulation and Fibrinolysis, and Early Coronary Stent Thrombosis
Source: J Clin Med. 2024 Dec 26;14(1):56. doi: 10.3390/jcm14010056 (PMC11721602; doi:10.3390/jcm14010056)
Supplement: Supplementary file 1 [file jcm-14-00056-s001.zip › jcm-3360389-supplementary.pdf]

Supplemental Table S1. Baseline characteristics comparing patients with available pre-procedural platelet count, fibrinogen or D-Dimer to patients with no available biomarkers.

	Biomarkers not available (n = 4.377)	Biomarkers available (n = 6337)	p Value
Age, median (IQR)	63.67 (54.3-72.3)	63.63 (54.1-72.5)	* 0.046
**Gender**			0.804
Male, n (%)	3,164 (72.3%)	4567 (72.1%)	-
Female, n (%)	1,213 (27.7%)	1770 (27.9%)	-
BMI, kg/m^2, median (IQR)	27.1 (24.6-30)	27.4 (24.7-30.7)	* <0.001
Smoker, n (%)	1,972 (45.1%)	3.053 (48.2%)	* 0.001
Diabetes mellitus, n (%)	1,040 (23.8%)	1583 (25%)	0.149
Hypertension, n (%)	2739 (62.6%)	4529 (71.5%)	* <0.001
Atrial fibrillation, n (%)	129 (2.9%)	279 (4.4%)	* <0.001
Family history of CAD, n (%)	353 (8,1%)	1344 (21.2%)	* <0.001
Previous myocardial infarction, n (%)	1308 (29.9%)	1317 (20,9%)	* <0.001
Previous PCI, n (%)	1,049 (24%)	1091 (17.3%)	* <0.001
Previous CABG, n (%)	531 (12.1%)	498 (7.9%)	* <0.001
Total cholesterol (mg/dl), median (IQR)	176.5 (155-209.8)	193 (161-229)	* <0.001
HDL (mg/dl), median (IQR)	44 (37-53)	43.5 (36-52)	0.235
LDL (mg/dl), median (IQR) †	107.5 (74.8-134.8)	109.5 (84-138.8)	0.641
Triglycerides (mg/dL), median (IQR)	150 (101-203)	135 (95-200)	0.785
HbA1c (%), median (IQR)	5.9 (5.6-6.9)	5.9 (5.5-6.4)	0.488
Creatinine (mg/dl), median (IQR)	1.09 (0.94-1.41)	1.05 (0.91-1.24)	* 0.045
**Indication for PCI**			* <0.001
SIHD, n (%)	3981 (91%)	3174 (50.1%)	-
ACS, n (%)	396 (9%)	3163 (49,9%)	-
**ACS type (n = 3,124/n = 43)**			* < 0.001
STEMI, n (%)	339 (85.6%)	1663 (52.6%)	-
NSTEMI, n (%)	19 (4,8%)	1007 (31.8%)	-
Unstable angina, n (%)	38 (9.6%)	493 (15.6%)	-
Number of stents implanted, mean ± SD	1.38 (±0.823)	1.57 (±1.058)	* < 0.001
**No. of coronary vessels diseased**			* 0.004
Single vessel disease, n (%)	1,883 (43%)	2,881 (45.5%)	-
Multivessel disease (>1 vessel), n (%)	2.494 (57%)	3,456 (45.5%)	-

AP, angina pectoris; ACS, acute coronary syndrome; CABG, coronary artery bypass graft; BMI, body mass index; SIHD, stable ischemic heart disease; HDL, high-density lipoprotein; LDL, low-density lipoprotein; STEMI, ST-elevation myocardial infarction; IQR, in-terquartile range; PCI, percutaneous coronary intervention; SD, standard deviation; ST, stent thrombosis. † LDL value calculated with Friedewald formula; * p ≤ 0.05 = significant;

Supplemental Figure S1: Kaplan-Meier curves for early stent thrombosis according to quintiles of coagulation parameters in patients with stable ischemic heart disease (SIHD)

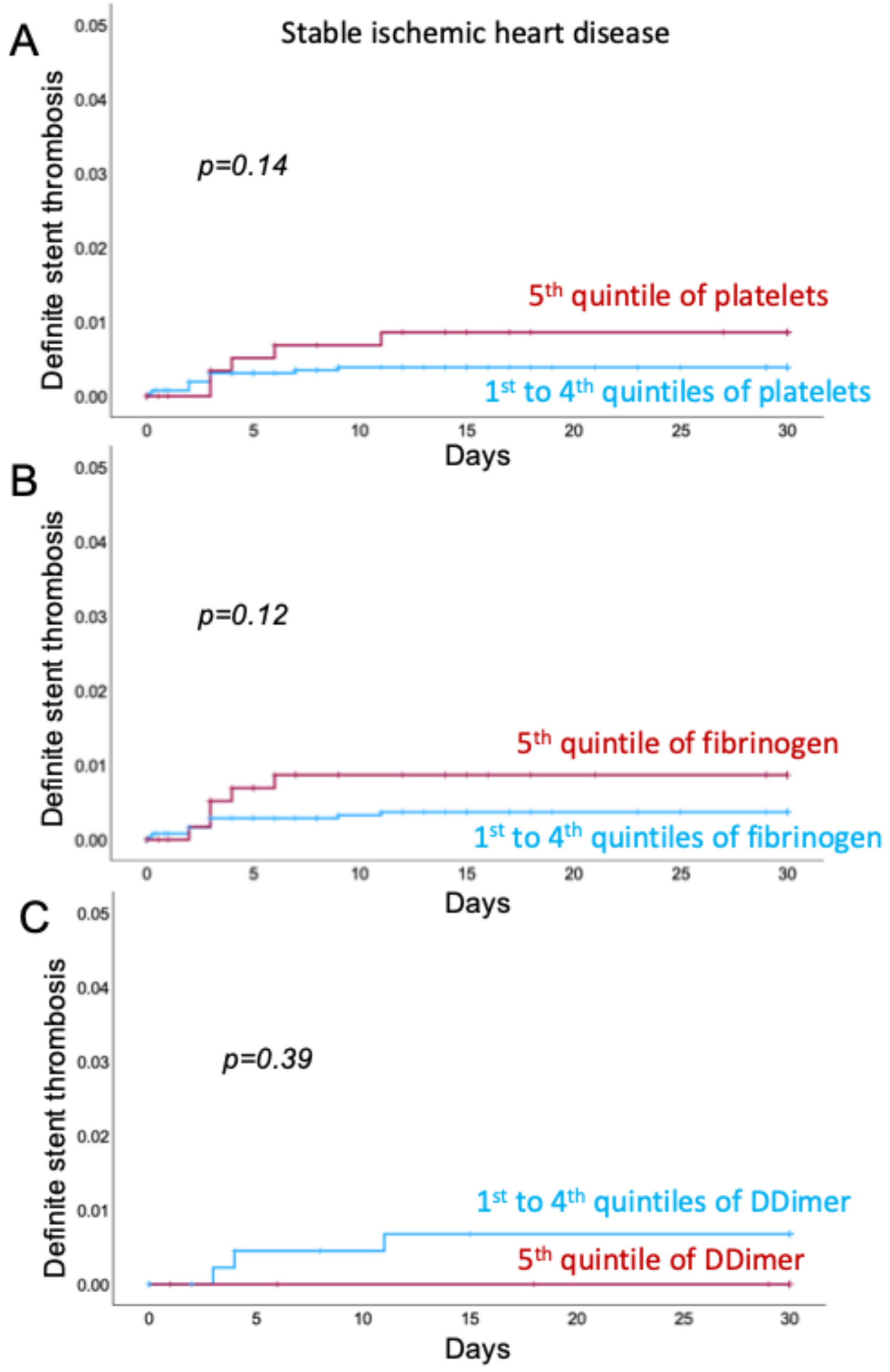

Patients were stratified according to pre-procedural platelet count (A), plasma levels of fibrinogen (B) and D-Dimer (C) above and below the 5th quintile. The p-values were calculated by log-rank test.

Supplemental Figure S2: Kaplan-Meier curves for early stent thrombosis according to quintiles of coagulation parameters in patients with acute coronary syndrome (ACS)

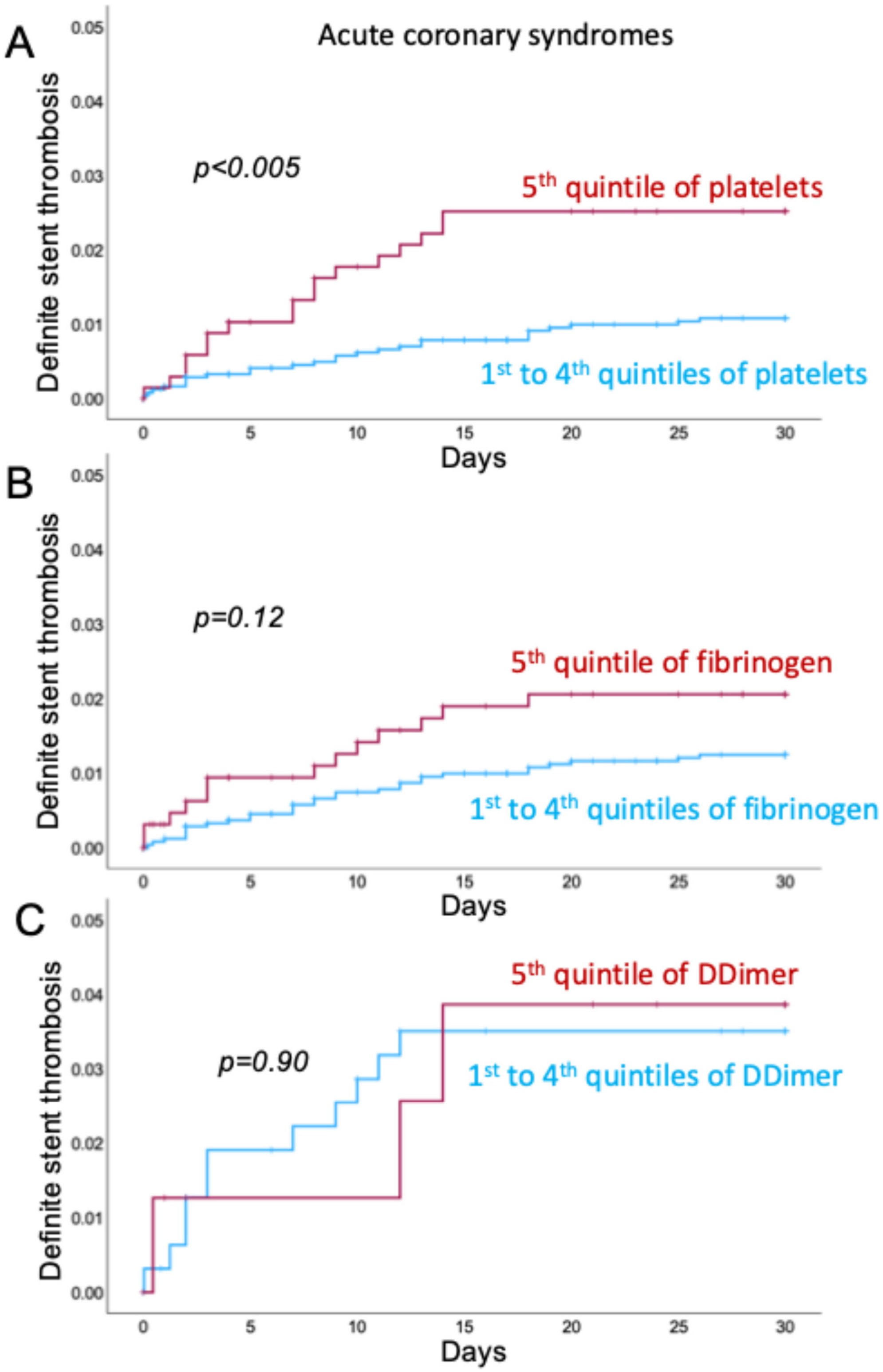

Patients were stratified according to pre-procedural platelet count (A), plasma levels of fibrinogen (B) and D-Dimer (C) above and below the 5th quintile. The p-values were calculated by log-rank test.

Supplemental Figure S3: Kaplan-Meier curves for early stent thrombosis according to cut-off values for pre-procedural platelet count and fibrinogen

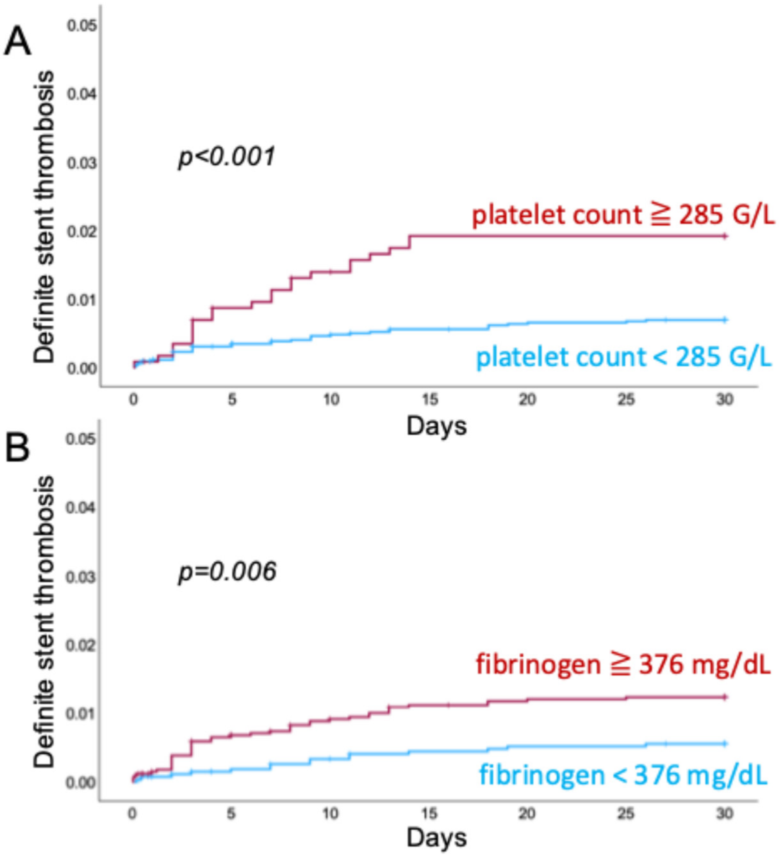

Patients were stratified according to cut-off values for pre-procedural platelet count (A) and plasma levels of fibrinogen (B). The p-values were calculated by log-rank test.

Supplemental Figure S4: Precision-recall curves for platelet count and fibrinogen

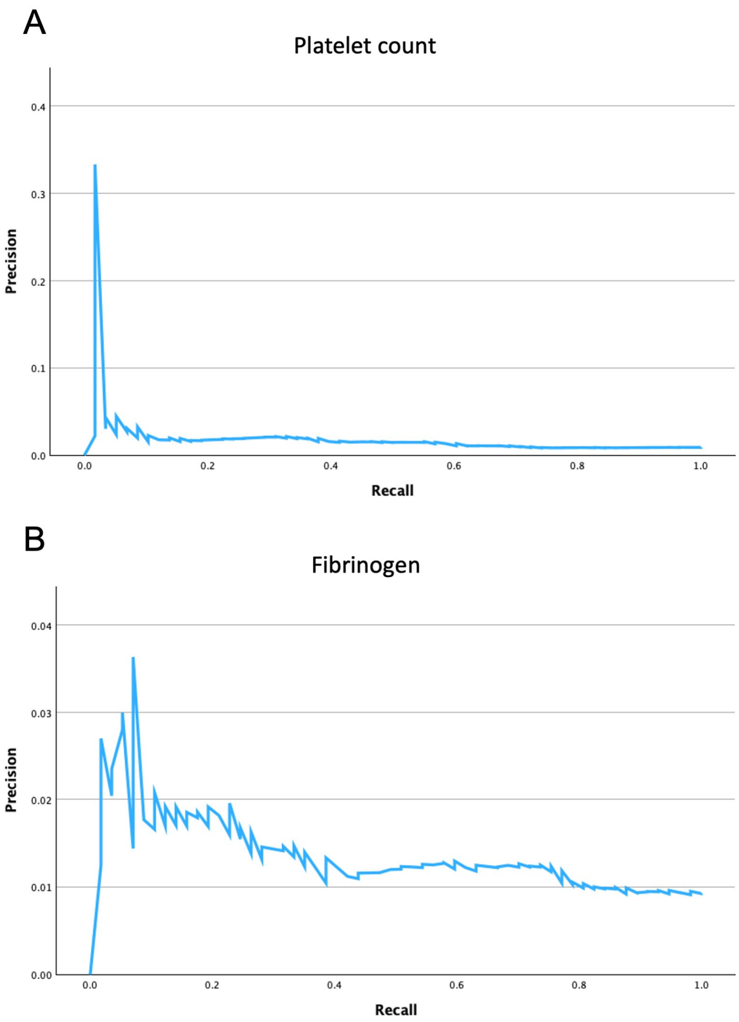

Precison-recall curves were calculated for pre-procedural platelet count (A) and fibrinogen values (B).

Supplemental Figure S5: Kaplan-Meier curves for survival and early stent thrombosis according to quintiles of coagulation parameters in patients undergoing percutaneous coronary intervention (PCI).

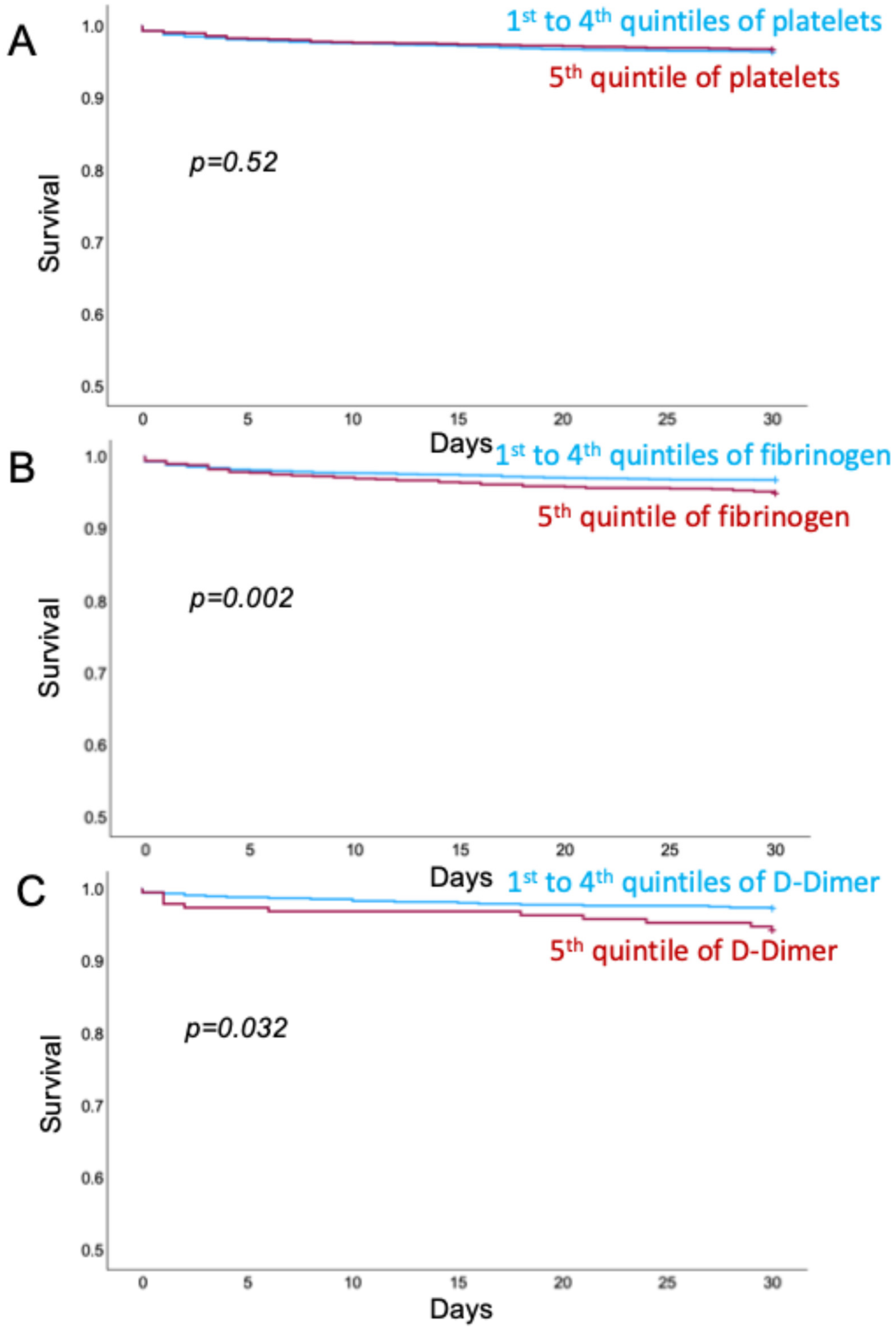

Patients were stratified according to pre-procedural platelet count (A), plasma levels of fibrinogen (B) and D-Dimer (C) above and below the 5th quintile. Kaplan-Meier curves were calculated for total mortality. The p-values were calculated by log-rank test.
